# Antibacterial Surface Treatment for Orthopaedic Implants

**DOI:** 10.3390/ijms150813849

**Published:** 2014-08-11

**Authors:** Jiri Gallo, Martin Holinka, Calin S. Moucha

**Affiliations:** 1 Department of Orthopaedics, Faculty of Medicine and Dentistry, Palacky University Olomouc, University Hospital, I. P. Pavlova 6, Olomouc 77520, Czech Republic; E-Mail: MHolinka@seznam.cz; 2 Department of Orthopaedic Surgery, the Mount Sinai Joint Replacement Center, Icahn School of Medicine at Mount Sinai, 5 E. 98th St., New York, NY 10029, USA; E-Mail: calin.moucha@mountsinai.org

**Keywords:** orthopaedic, biomaterial-associated infection, prosthetic joint infection, anti-adhesive, antibacterial, surface treatment, silver, antibacterial proteins, smart surfaces

## Abstract

It is expected that the projected increased usage of implantable devices in medicine will result in a natural rise in the number of infections related to these cases. Some patients are unable to autonomously prevent formation of biofilm on implant surfaces. Suppression of the local peri-implant immune response is an important contributory factor. Substantial avascular scar tissue encountered during revision joint replacement surgery places these cases at an especially high risk of periprosthetic joint infection. A critical pathogenic event in the process of biofilm formation is bacterial adhesion. Prevention of biomaterial-associated infections should be concurrently focused on at least two targets: inhibition of biofilm formation and minimizing local immune response suppression. Current knowledge of antimicrobial surface treatments suitable for prevention of prosthetic joint infection is reviewed. Several surface treatment modalities have been proposed. Minimizing bacterial adhesion, biofilm formation inhibition, and bactericidal approaches are discussed. The ultimate anti-infective surface should be “smart” and responsive to even the lowest bacterial load. While research in this field is promising, there appears to be a great discrepancy between proposed and clinically implemented strategies, and there is urgent need for translational science focusing on this topic.

## 1. Introduction

Biomaterial-associated infection is a disastrous complication of modern orthopaedic surgery that often leads to prolonged patient pain and functional losses. While international efforts to minimize the risk of these infections are underway [1], orthopaedic surgical site infections (SSIs) continue to occur in staggering numbers. Current estimates suggest that up to 2.5% of primary hip and knee arthroplasties and up to 20% of revision arthroplasties are complicated by periprosthetic joint infection (PJI) [2]. According to some authors not only are these numbers underestimates but they are also on the rise [3]. *Staphylococcus aureus* is the leading cause of both the SSIs and PJIs, and the prevalence of methicillin-resistant *S. aureus* (MRSA) SSI and PJI is increasing, especially in the United States [4]. Generally, deep infection leads to implant removal and ensuing increased morbidity and even mortality [5]. Moreover, therapy of PJI is associated with enormous costs [6].

Although methods developed for perioperative infection prevention such as antibiotic prophylaxis have been shown to be effective in SSI reduction, most assume a uniform intraoperative environment [7]. As the majority of operating rooms are contaminated within the first few hours of service [8,9], most surgeries are not performed in a bacterial-free environment. Within a certain operating room all patients are exposed to the same environment. The question therefore arises as to why some patients go on to have infections and others do not. This question has recently been re-examined; it is still premature, however, to give strict recommendations for clinical practice [10,11,12,13]. Even though modifiable SSI risk factors have been identified and well-described [7,14,15] it is not often possible to avoid operating on patients who are not “optimized”.

Several recent scientific forums have recommended that researchers should focus on the development of effective antibacterial surfaces that prevent bacterial adhesion, colonisation and proliferation into the surrounding tissues [1]. The aim of this review is to summarize current knowledge in this field with particular emphasis on technologies that could be suitable for prevention of PJI in total joint arthroplasty. Similar technologies could be employed for prevention of SSIs in other orthopaedic cases involving implants such as plates, intramedullary nails, and external fixators.

### 1.1. How to Win the Race for the Surface?

Gristina proposed the concept of a “race for the surface” whereby host and bacterial cells compete in determining the ultimate fate of the implant [16]. Accordingly, when host cells colonize the implant surface first the probability of attachment of bacterial cells is very low and vice versa. This concept has stimulated technological and biomaterial progress while emphasizing the role of implant biocompatibility and tissue-integration. This model, however, can be criticized for its simplicity (simple rules, assumptions *etc.*), static conditions, and low capacity for prediction of PJI (inability to help with quantification of clinical uncertainty). Specifically, it is not able to interpret a wide zone often found between basic polar items, *i.e.*, complete host cell *versus* bacterial cell coverage of an implant surface.

**Figure 1:**
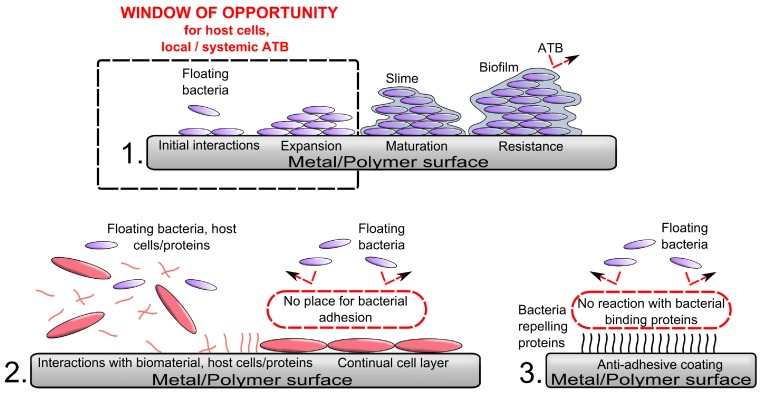
Schematic illustration of the process of biomaterial colonization starting from individual bacteria adhesion across micro-colonies towards formation and maturation of biofilm (1); Bacteria cannot activate the biofilm-related phenotype before they firmly attach to the substrate. After attachment and change in the phenotype they are able to produce the matrix of extracellular polymeric substances that protect them against host immune response and antibiotics. If host cell achieves irreversible attachments on the biomaterial surface first (*i.e.*, if they are the winner) it is difficult for bacterial cells to start with biofilm formation (2); The period before firm attachment and phenotypic change is therefore the window of opportunity for almost all antibiofilm strategies (3). During that time these strategies compete with bacteria for implant surface attachment and microenvironment. ATB = antibiotics.

The most destabilizing factor is the basic yet highly successful survival strategy of bacteria in general: their ability to adhere and survive on virtually all natural and synthetic surfaces [17,18]. Bacterial cell membranes contain various types of adhesins for a wide range of biomaterial surface receptor sites. Environmental and surface characteristics of a biomaterial such as surface roughness, hydrophobicity, and electrostatic charge play only conditional roles [19]. A reservoir of receptors for bacterial adhesive ligands mediating adhesion of free-floating bacteria to the surface of the biomaterial offers a conditional protein film covering an implant immediately after its placement into the host body [20,21,22,23]. Complement and albumin are considered the main components of this conditional protein film [24]. However, the protein spectrum extends much beyond complement and albumin and depends at least in part on a particular type of biomaterial attracting an exact set of host proteins and lipids [25,26,27]. Conceptually, the process of bacterial adhesion can be divided into two basic phases: reversible and irreversible (Figure 1) [28,29]. The former is mechanically and biologically less stable than the latter. The explanation lies in part on the origin of nonspecific interactions between implant surface characteristics and bacterial surface adhesins. The second phase is mediated by molecular and cellular interactions closely associated with expression of biofilm specific gene clusters in reversibly attached bacteria [30]. At least four distinct classes of surface proteins have been identified to participate on firm adhesion of *S. aureus* micro-colonies to a biomaterial and to each other [31]. An adhesion phase is followed by gene expression for secretion of protective slime. This process makes bacteria extremely resistant to both host immune system and antibiotic diffusion [29,32]. The transition between reversible and irreversible phases of biofilm formation coupled with phenotypical change is the last window of opportunity for clinically reasonable preventative measures.

On the host site, the details of tissue integration of a biomaterial are still poorly understood [33,34,35,36]. It is believed that host cells attached to implant fixation surfaces orchestrate the processes leading to periprosthetic bone regeneration and remodelling that protect against bacterial colonization [37]. However, neither osseointegration nor fibrous tissue encapsulation of large non-fixation parts of an implant can eliminate long-term survivorship of bacterial micro-colonies. Moreover, peri-implant fibrous barriers can prevent contact between host immunity sentinel cells and bacterial molecules. This interaction is critical for host immune responses dependent on recognition of bacterial pattern-recognition receptors (PRRs; also microbe associated molecular patterns = MAMPs). This cascade goes on to intracellular signal transduction by first-line cell adaptors that organize the appropriate host response via particular modules of innate and adaptive immunity [38]. Additionally, it has been demonstrated that implantation of a medical device impairs innate local host response and may facilitate the development of PJI [39,40,41]. As a result, there is a strong need for intrinsic implant surface antibacterial functionality that can overcome implant-induced defects in the local immune response. This is of utmost importance especially in patients with underlying compromised immunity [42] and in those undergoing revision surgery [42,43].

### 1.2. Brief Overview of Basic Concepts of PJI Prevention

Strategies relying on decreased bacterial load and creating bacteria-free environment around an implant during the perioperative period are widely implemented in clinical practice [44,45,46,47]. There is sufficient evidence supporting systemic and in some cases local antibiotic prophylaxis [48,49,50]. Attempts at formulating evidence-based standards for good clinical and logistic practice in orthopaedic operating rooms have been utilized [7,51,52,53]. Finally, while there is certainly room for improvement, educational programs aimed at educating orthopaedic surgeons (the entire staff) in perioperative strategies of PJI prevention are under way.

### 1.3. Indications for Implants with Antibacterial Surface Treatment

An important consideration in designing implants with antibacterial coating relates to the characterization of reasonable and justifiable cost [54]. Theoretically all patients undergoing total joint arthroplasty are at risk for PJI. Revision cases carry an increased risk in part due to the suboptimal local tissue environment [43,55,56,57]. Moreover, several studies emphasize that the risk of PJI across the board in orthopaedic surgery is on the rise [3,58,59]. As a result, one could argue that all patients should benefit from implants coated with a proven anti-infective surface. On the other hand, the risk for PJI is not homogenously distributed among the arthroplasty patients: it is stratified into the specific groups [42,60,61,62,63]. Therefore, it might be convincing to implant “biofilm resistant” prostheses only in patients at increased risk of PJI. A validated tool for screening patients for increased risk of PJI does not currently exist. Despite attempts to identify and stratify patients at risk of PJI [12,42,64,65,66] specific clinical algorithms are not routinely used. In addition, we have no data relevant for determining the potential costs associated with wide range usage of such a screening strategy. Taken together, the preventative strategy involving all patients undergoing primary and revision total joint arthroplasty seems to be more justifiable than a more restrictive approach targeting high risk patients. However, prior to implementation of such devices, it is necessary to demonstrate the significant reduction of PJI in a well-done population-based cost-benefit analysis [37].

### 1.4. Rules for Construction of Implants with Anti-Infective Coating

A wide spectrum of substances and technological approaches has been proposed and tested for antibacterial features (Table 1). In order to fully discuss and evaluate surface treatment technologies it is essential to review strict criteria related generally to the process of innovation in this field. These are as follows: (i) biocompatibility (the ability of a material to perform with an appropriate host response in a specific applications) [67]; (ii) strong evidence of anti-infective efficiency (the anti-bacterial efficiency should be demonstrated *in vitro*, *in vivo* and also in an appropriate model of PJI) [68]; (iii) fixation properties cannot be compromised (the antibacterial coating must not compromise long-term stable implant osseointegration or cement fixation); (iv) durability of the anti-infective effect (while clear recommendations are lacking epidemiological viewpoints suggest that at least two years would be appreciated) [55,58]; (v) mechanical characteristics of the antibacterial coating (resistance to mechanical stresses and strains either during surgery or postoperatively).

**Table 1:** Examples of anti-infective strategies proposed for treating of surfaces used in orthopaedic surgery.

Strategy	Features	Examples	References
Prevention in adhesion and adsorption		Anti-adhesive polymers	[68,69,70,71]
		Albumin	[72]
		Super-hydrophobic surfaces	[73,74,75]
		Nano-patterned surface	[76,77,78,79]
		Hydrogels	[80,81,82,83]
Methods to kill bacteria	Inorganic	Silver nanoparticles	[84,85,86,87,88,89,90]
		Titanium dioxide	[91,92,93]
		Selenium ion	[94,95,96]
		Copper ion	[97,98]
		Zinc ion	[99,100]
	Organic	Coated or covalently linked antibiotics	[101,102,103,104,105]
		Chitosan derivatives	[106,107,108,109]
		Signaling, inhibiting and antimicrobial peptides	[110,111,112,113,114,115]
		Cytokines	[116]
		Enzymes	[117,118]
	Other	Non-antibiotic bactericidal substances	[119]
	Combined	Multilayer coating	[120,121,122,123,124]
		Synergy material intensification	[125]
		Positively charged polymers	[126]
Multi-functional and smart coating	Passive	Nanostructured “smart” material	[71,127,128]
	Active	Concept: sensors conjoined to nanocontainers	[129,130,131,132,133]
Alternative approach		Lytic bacteriophages	[134]

### 1.5. General Principles of Thin Surface Modifications

A change in the surface chemistry and/or structure of the bulk implant can be achieved either by chemically or physically altering the surface layer in the existing biomaterial (e.g., oxidation or mechanical modifications like roughening/polishing/texturing). A different method involves over-coating the existing surface with a new thin layer of material having a different composition (e.g., hydroxyapatite coating on titanium alloys, antibiotics bound covalently to the substrate, fixation of other antimicrobial compounds) [135]. In terms of durability, we can distinguish between degradable and non-degradable biomaterials [136].

### 1.6. Remarks on the Testing of Antibacterial Coatings

A critical step in progress lies in the demonstration that newly developed biomaterials possess antibacterial efficacy [137]. To date there is no widely accepted methodology available that could precisely and reproducibly demonstrate antibacterial behaviour of the proposed anti-infective technologies. Major criticisms lie around static “closed” testing system whereas *in vivo* the implant has to face a dynamic, continuously changing, mechanically unstable and predominantly fluid environment [138]. As a result, the majority of studies to date have used inappropriate and insufficient protocols.

Controllable, standardized testing conditions that closely mimic the human *in vivo* environment are needed in order to overcome the aforementioned issues [138]. PJIs develop at low shear conditions and under multidirectional low-pressure fluid flow. A variety of testing tools have been proposed that attempt to simulate conditions of continuous or intermittent fluid-displacement in both low and high shear conditions [139]. Protocols for cultivation of particular species (multispecies) biofilms at controllable, constant and reproducible conditions have also been described [140]. Finally, representative *in vitro* and *in vivo* models for each particular clinical situation (*i.e.*, total joint arthroplasty, internal, external fixation) should be further developed and appropriately validated. Given the large variability of antibacterial strategies it is likely that testing methods must be better tailored to match the specific proposed strategy at hand [141].

## 2. Basic Concepts of Antibacterial Coating

**Figure 2:**
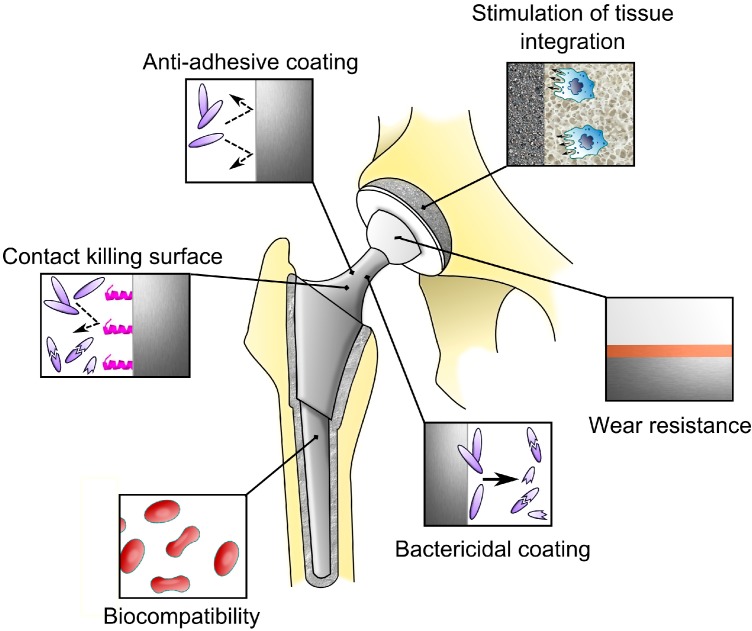
Ideas of multifunctional surfaces in total hip arthroplasty designed to simultaneously or successively respond to various biological and mechanical tasks. The response depends on the specific abilities of the coatings acquired during the manufacturing process.

A number of principles from basic research have been proposed for translation into technologies potentially suitable for antibacterial treatment of orthopaedic implants. It is currently easy to distinguish between technologies offering anti-adhesive properties, those working as antimicrobial agents, and those combining above-mentioned approaches. Anti-infective surfaces can be classified as “contact killing” and antimicrobial agent eluting [141]. Coatings can also be degradable and non-degradable. In terms of functionality, one may choose to divide surfaces into mono-functional and multi-functional. The latter are expected to target multiple biological tasks simultaneously (Figure 2). A “smart surface” is a completely different methodology designed to be a self-responsive multitask micro-machine that releases antimicrobial (and other) substances after stimulation by microbiological (or other) signals.

### 2.1. Anti-Adhesive Approaches

Engineers believe they are able to treat biomaterial surfaces in this manner and that this will prevent the critical step of bacterial biofilm formation. As a result, such a strategy could potentially target infections related to perioperative contamination of prosthetic surfaces [142]. Some authors believe that hydrophilic, highly hydrated, and non-charged surfaces could be a good choice. These surfaces have been shown *in vitro* to prevent many bacterial species from biomaterial adhesion by limiting the contact between bacterium and potential surface placement sites [143]. Host cells attachment, however, may also be negatively affected by certain surface treatments. While such strategies cannot be used in the setting of implants requiring bony ingrowth (such as fixation surfaces in cementless arthroplasty implants) they may be appropriate for non-fixation surfaces (plates, screws, or intramedullary nails). In addition, it should be mentioned that the basic concept favouring the hydrophilic over the hydrophobic forces might be criticised from both the bio-physicochemical misunderstanding of these terms and the complexity of biological interactions around an implant [75,138,144,145,146]. As a result, much more attention has been focused recently on hydrophobic and superhydrophobic surface treatment technologies and their repellent antibacterial effects [73,147].

**Figure 3:**
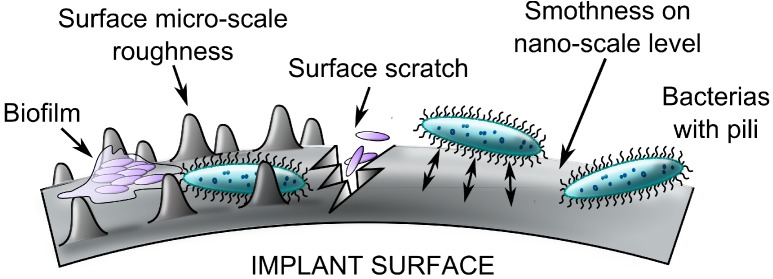
The relationship between biomaterial surface roughness/chemistry and bacterial attachment is intricate. Bacterial attachment is facilitated by increased surface microscale roughness since larger surface areas (especially when irregular) provide binding sites and protection. Increased smoothness of the surface should prevent bacterial colonization. A similar scenario is found with corrosion associated surface micro-cracks that are prone to infection. On the other hand, exceptionally smooth materials can increase the bacterial attachment via physical forces such as van der Waals interactions (double rows) and by providing a number of molecular contact points.

Treating protein-surfaces and/or protein-bacteria interactions may be a good strategy of inhibiting bacterial adhesion to a specific biomaterial [142]. Proteins such as albumin, fibronectin, fibrinogen, laminin, denatured collagens, and some plasma/tissue lipids are the first host substances that interact with the surface structure of the biomaterial [21,27,148]. Reduction of conditional lipid-protein layer formation can be achieved by changing surface physico-chemical characteristics, and/or surface micro-morphology [149]. In fact, a number of studies have demonstrated that the biological response to biomaterials can be controlled via alterations in surface chemistry and structure [22,35,150,151]. For instance it has been suggested that implants with rough and porous surface structure are prone to greater bacterial adhesion in comparison to smooth surfaces. This is perhaps due to much larger surface area available for adhesion and subsequent higher number of anchor points [152]. Porous cementless implants have much larger surface available for bacterial adhesion; some studies report their usage is associated with increased risk of infection as compared to cemented ones [153]. However, other investigators found similar risk for infection in relation to the type of total hip arthroplasty fixation [154]. This could point paradoxically to the complexity of the clinical situation where a myriad of factors participate in PJI pathogenesis and surface roughness is only one of many, implant related characteristics. At the nanometre scale, bacterial adhesion does not simply follow the roughness of the surface but also is dependent on other variables like the quantity of adsorbed proteins. When roughness increases, the formation of a thick protein layer on such implant surface could suppress bacteria adhesion [77]. In addition, the adhesion process can be different among the materials with different surface structure in terms of short-range van der Waals interactions and surface energy (Figure 3), [19,155,156,157,158].

To date a number of anti-adhesive tactics have been proposed for different purposes. Only a few, however, have met the elementary features required for bone implant usage. Friedman *et al.* using a rabbit model, demonstrated reduced bacterial adherence on pure titanium samples and decreased infection rates of implants coated with cross-linked albumin [72,159]. Surprisingly, this model has not been further pursued. More recent strategies include production of self-assembled mono- or multilayers, surface grafting, or hydrogels [142]. Importantly, the level of anti-adhesive properties has to respect the purpose of a particular type of orthopaedic implant surface (*i.e.*, whether surfaces are intended for total joint arthroplasty, internal fixation, external fixators). Specifically, a strong anti-adhesive layer cannot be used for coating of fixation surfaces of total joint arthroplasty because it could also prevent host bone osseointegration and lead to early mechanical failure. The solution lies in a coating technology that retains required host cell interactions while selectively inhibiting bacterial adhesion. [160]. It was found that specific changes of a surface morphology at the micro- and nanometre scales might influence not only the bacterial adhesion but also biofilm phenotype conversion [155,157,161,162,163]. As a result, nanopatterning and other surface treatment nanotechnologies can offer new opportunities for development of effective anti-adhesive treatment in orthopaedic implants [156,164].

Taken together, anti-adhesive technologies offer attractive opportunities for engineers and collaborating researchers to develop a prosthetic surface that should ultimately diminish PJI rates. This approach does however have some important limitations. Cementless arthroplasty implants that require host bone integration may not be amenable to such coatings. The process of unifying ongrowth or ingrowth with antibacterial anti-adhesive functionality as part of a surface coating is technically demanding and has not been fully elucidated. Another challenge of designing antiadhesive technologies relates to the current inability to design a universal surface treatment that can be applied to all surfaces, all bacterial species, and under all (ingrowth and noningrowth) implants.

### 2.2. Surfaces with Intrinsically Antibacterial Properties

**Figure 4:**
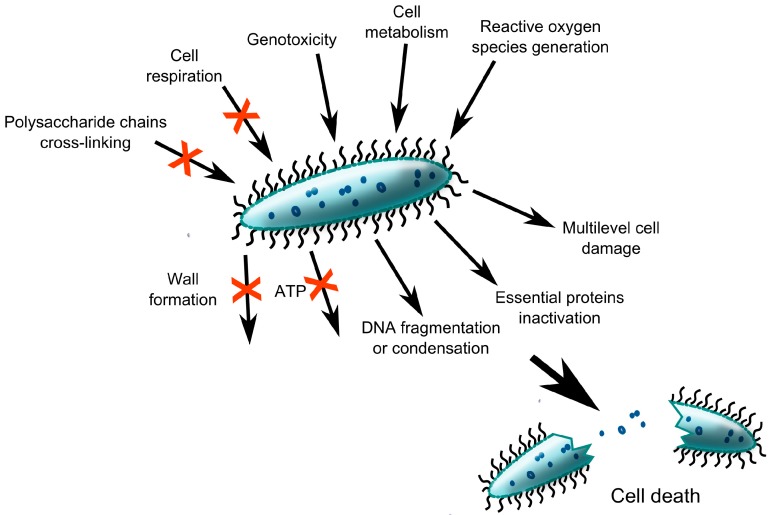
Potential mechanisms by which antibacterial substances kill bacteria; critical targets include bacterial cells genome, metabolic and respiration pathways, cell envelope synthesis and membrane disruptors (e.g., reactive oxygen species).

Historically, two main strategies have been proposed for effective antibacterial surface treatment either “contact killing” or drug eluting. The majority of them are not suitable for surface treatment of orthopaedic implants due to problems with cytotoxicity, immunoreactivity, and genotoxicity [165,166,167,168]. In killing bacteria they rely on diverse mechanisms of action, which may interfere with a cell respiration, cell division, or formation of a cell wall (Figure 4). Another very promising approach involves interference of the bacterial signalling network (e.g., quorum sensing) or inhibition of the transition of planktonic phenotype of bacteria into a sessile type [138]. This tactic could prolong the window of opportunity for both prophylactic antibiotic activity and the host immune response.

Antibacterial surface technologies can employ metals (silver, zinc, copper, zirconium *etc.*), non-metal elements (e.g., selenium), organic substances (antibiotics, anti-infective peptides, chitosan, other substances), and their combinations.

#### 2.2.1. Coating of Implant Surface by Anti-Infective Metals

Antibacterial activity of the majority of metal coatings is closely linked to the ionic or nano form rather than to the bulk material [169]. Despite extensive research, routine coating of implants with such a thin layer of metal is still not the standard [170]. The main obstacles preventing broader usage of such technologies are cytotoxicity and resultant decreased biocompatibility. In addition, creating a coating-substrate interface robust enough to sustain the mechanical stresses involved in surgical implant insertion and ultimate loading once *in vivo* remains a challenge [171]. Lastly, the risk of bacterial resistance to metallic coatings, a phenomenon common to all antibacterial strategies, remains a concern.

Silver is the most prevalent metal used in biomedical applications. Dissolved silver cations are biochemically active agents that interfere with bacterial cell membrane permeability and cellular metabolism. Silver also contributes to formation of reactive oxygen species and other mechanisms that potentially influence prokaryotic cells [172]. There has been concern, however, about the toxicity of silver ions. Even in minute levels silver can adversely affect surrounding cells and lead to potentially harmful accumulation in distant locations [173]. Research efforts have focused on the development of silver coating technologies that reduce or even eliminate toxicity while maintaining constructive antibacterial effects [174,175]. Panacek *et al.* showed that ionic silver inhibited the growth of the *Candida albicans* at concentrations comparable to levels (approximately 1 mg/L) that were cytotoxic against human fibroblasts. In contrast, silver nanoparticles (see Section 2.2.4.) effectively inhibited the growth of the tested yeasts at concentrations below the cytotoxic limit against human fibroblasts (30 mg/L) [176].

Copper and zinc are trace metals involved in multiple enzymatic and cellular processes. These metals also have potent antibacterial effects on a wide spectrum of bacterial species [169,177,178]. The ability of bacteria to survive around copper compounds depends on the expression of copper tolerance genes [179]. The same pathways are required for certain bacterial species to survive innate immune attacks. Potential toxic side effects of these metals remain a strong concern [98,180]. Potential solutions may incorporate copper- and zinc-based nanomaterials or, alternatively, more controlled release of these metals in conjunction with other surface infection control strategies [181,182].

Cobalt-chrome and titanium alloys are the most commonly used materials in total joint arthroplasty implants. Several technologies have been proposed to expand the antibacterial properties of these implants [183]. Functionalization of biomaterial surfaces with silver and copper ions is one such method [171,175,184]. The anti-infective potential of titanium dioxide layers has also been widely investigated both alone [185,186] or in combination with other substances [100,187]. This concept has been tested in external fixator pins that are particularly prone to infection [91,92]. In one study, silver coated pins were compared to uncoated ones. While the rate of bacterial colonization was slightly lower in the silver coated pins, the differences were not significant. More importantly, the patients with silver coated pins exhibited a significant increase in serum silver levels and the study required termination [188]. Great expectations are associated with polyetheretherketone (PEEK) implants. These implants could become immune to bacterial colonization by employing the chelate-bonding ability of inositol phosphate to immobilize silver ions on the hydroxyapatite film of the PEEK substrate [189]. Such implants are not currently available in the joint arthroplasty field.

#### 2.2.2. Non-Metal Elements with Antibacterial Properties for Implant Surface Treatment

Non-metal elements like hydrogen, chlorine, iodine, or oxygen are commonly used in biomedicine for their anti-infective properties. They have been rarely indicated as antibacterial coating technologies in orthopaedic implants due to their general softness and brittleness. Selenium bound covalently onto the surface of titanium or titanium alloy implant discs have been shown to prevent *Staphylococcus aureus* and *Staphylococcus epidermidis* attachment without affecting osteoblast viability [94]. Selenium catalyzes the formation of superoxide radicals and subsequently inhibits bacterial adhesion and viability. In addition, selenium nanoparticles can inhibit bacterial growth and biofilm formation [95,190]. Ongoing research is needed to determine the clinical applicability of carbon substances like graphene or carbon nanotubes that can be synthesized in multifunctional layers [191].

#### 2.2.3. Antibacterial Coatings of Organic Origin

**Figure 5:**
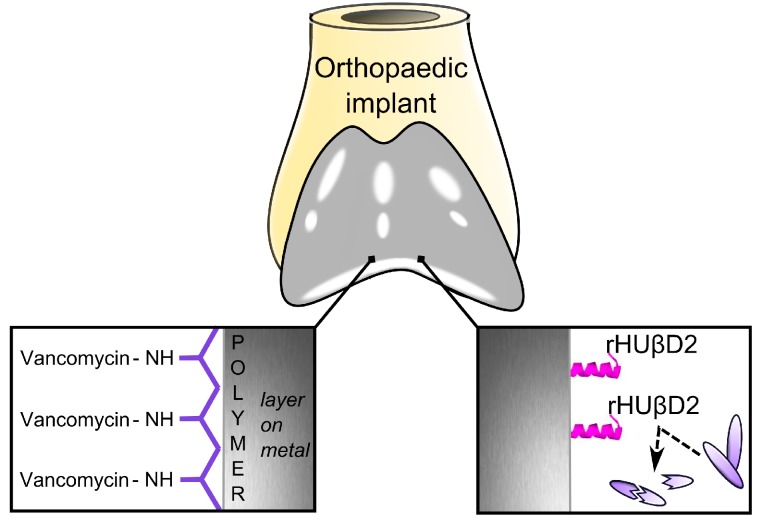
Two examples of antibacterial effect located on the surface of an implant. The first example demonstrates Vancomycin linkage to a carrier polymer; the second example shows tethered cationic antimicrobial peptides creating a contact killing surface utilizing recombinant human β-defensin-2 (rHUβD2).

A large number of studies have investigated the efficacy of surfaces coated with covalently linked antibiotics (Figure 5), [102,192,193,194,195]. Clinical effectiveness of such implants is most likely limited to infections caused by bacteria that are sensitive to the specific antibiotic that has been coupled. In addition, strong forces such as covalent binding are insufficiently sensitive to react to weak external stimuli [133]. To overcome these issues, combinations of antibiotics with other compounds have been proposed either alone or in association with a particular mechanism of controlled release [196].

A promising new approach for prevention of implant-related infection involves coating implants with antimicrobial peptides, cytokines or other molecules critical for host response to bacteria invasion [116,119,142,197,198]. This heterogeneous group of substances has proven experimentally their efficacy against a wide range of pathogens [199]. They employ a number of mechanisms, pathways, and targets that participate in implant bacterial invasion including those related to local deficiency in immune response induced by surgical approach or the implant insertion method itself. Antimicrobial peptides, like antibiotics, function via damage of the cell wall and inhibition of key bacterial protein synthesis. In addition, they exert influence upon inflammation, tissue healing, and apoptotic events [200,201]. Notably, resistance to antimicrobial peptides has been reported less frequently than to antibiotics [202]. Initial experiments demonstrated that a thin layer of antimicrobial peptides affixed onto the surfaces of orthopaedic (dental) metal alloys exhibit excellent antibacterial effects against typical pathogens related to PJI [203,204,205,206,207]. In addition, it has been demonstrated in a rat model that an interleukin-12 nanocoating substantially decreased infection rates [116,208]. Other immunomodulatory proteins such as chemokines can also exhibit antimicrobial activities [199]. The possibility of multifunctional layers (targeting both the tissues homeostasis maintenance and infection) may become reality.

Recently, molecules and compounds that interfere with the expression of various bacterial phenotypes have shown great promise [114,209,210,211,212]. In the field of PJIs, weapons aimed at Staphylococcal bacteria are the most valuable. In this line, the autoinducer two (AI2) signaling peptides, RNAIII-inhibiting peptide (RIP), or dihydropyrrolones might be potential candidates for total joint arthroplasty surface treatment [113,213,214,215]. A very promising set of new molecules called biofilm disruptors has been discovered recently [216]. They might not only protect an implant surface from biofilm formation but also disrupt existing biofilms. However, in contrast to local delivery of antibiotics, the optimal doses and surface pharmacokinetics of above-mentioned substances need to be determined. To our knowledge, we are not aware of any orthopaedic experimental models that test these aforementioned technologies.

Chitosan (CS) is a polycationic polymer derived from chitin that exhibits antibacterial and antifungal activity. The exact mechanism of action remains poorly understood. Some studies found that macrophages are more effective when working on chitosan surface [217]. Derivatives of CS have recently been widely studied in relation to antibacterial usage in biomedicine. One such compound, quaternized CS, has shown strong antibacterial activity against both Gram-positive and Gram-negative bacteria [109]. The mechanism of action involves bacterial surface adhesion deterrence as well as inhibition of transcription factors required for production of extracellular matrix [218,219]. There is some evidence that CS derivatives can be firmly anchored to titanium alloys and that they have a protective effect against some bacterial species either alone or in combination with other antimicrobial substances like antibiotics or antimicrobial peptides [112,218,220,221,222]. CS derivatives secured to external fixator pins have been studied as a method of preventing pin tract infections [223]. However, we are not aware a study to date reporting data from clinical setting. Several studies also investigated the potential of CS derivatives in the form of nanoparticles to protect bone cement (polymethylmethacrylate) against bacterial colonization and biofilm formation [219,224].

#### 2.2.4. Nanostructured Surfaces and Coatings

Nanostructured surfaces and coatings (either inorganic or organic origin) are currently of great interest [225,226,227]. Consequently, nanoscale surface patterning methods have been applied to fabricate different nanopatterns (e.g., ordered stripes, pits, pillars or squares). Several studies have demonstrated that nanopatterning in conjunction with other surface treatment could inhibit bacterial adhesion [101,157,228,229,230].

Another example of nanotechnology application is fabrication of polymers containing antibacterial nanoparticles and substances that inhibit both the quiescent and sessile bacteria [231]. Synthetic polymers, natural polymers, and their derivatives (e.g., gelatin, chitosan) have potential to be used as implant surface scaffolds and delivery vehicles of antibacterial agents [82,232,233,234,235].

**Figure 6:**
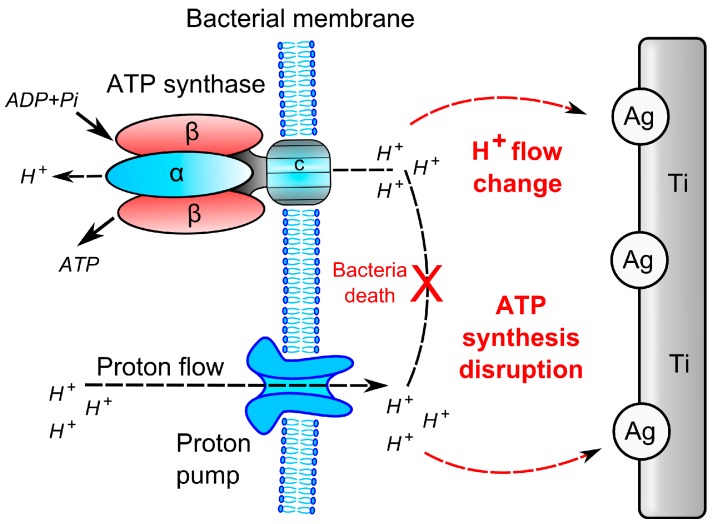
Plasma-surface modification techniques creating a special type of antimicrobial surface involving silver (Ag) nanoparticles embedded into titanium (Ti); it is proposed that Ag and Ti represent a micro-galvanic pair with different potentials in the presence of electrolyte solution; the cathodic reaction will create a proton-depleted region between bacterial membrane and Ti substrate, which leads to disruption of the adenosine triphosphate synthesis and bacteria death.

The antibacterial effect of silver nanoparticles (NP) is not fully understood to date. It might be based on the release of silver cations from nanostructured surfaces (Figure 6). These cations permanently disrupt bacterial cell wall, inactivate essential proteins, cause DNA condensation, and lead to reacting oxygen species generation [90,125,236]. The antibacterial activity of the silver NPs is dependent on both size and shape. Differences in the mechanism of action of diverse forms of silver may explain why to date there have been no reports of resistance to this type of antibacterial treatment [89,235]. As compared to non-nanoscale silver applications, a nanoscale form offers simultaneously greater solubility, chemical reactivity, and strong antibacterial activity even at low concentrations (units of milligrams per liter), [176,237,238]. Silver NPs have been shown to cover a wide spectrum of causative bacteria [239,240]. Moreover, *in vitro* and *in vivo* experiments have shown long-lasting antibacterial protective effects of nanostructured titanium coating incorporated with silver NPs [84,241]. Intense research is being done to combine both the antibacterial effect of silver NP with osteointegrative properties and improved biocompatibility of materials such as titanium alloys [87,187,242,243,244]. Lastly, nanoparticles of selenium, copper, zinc, and other elements have also demonstrated strong antibacterial efficacy [245,246,247].

Taken together, nanotreatment of biomaterial surfaces offers new opportunities for periprosthetic joint infection prevention. Early studies have shown high biocompatibility of such approaches and therefore great potential for use in surface treatment of orthopaedic implants [107,225,248,249,250]. It should be cautioned, however, that nanotechnologies can also induce unintended inflammatory responses related to activation of dendritic cells and macrophages [251]. Concern also exists about the mechanical properties of implant nanocoatings since damage may occur during surgical implantation, especially in cementless implants inserted via press-fit methods [252]. Methods describing the nanosilver coating of medical device in orthopaedics and traumatology have been patented in both the United States and in the European Union. Implants covered by a nanosilver coating are not currently available in clinical practice. At least two manufacturers in Germany, however, already produce on request total joint arthroplasties treated by a galvanic deposition of elementary silver. Initial clinical experiences with these “custom made” implants are promising [253,254].

## 3. Multifunctional and Smart Coatings

Multifunctional surface layers have been developed in an effort to coalesce the need for implants possessing anti-infective properties (see Section 1.4.) with much needed maintenance of perioperative tissue homeostasis. Along these lines a functional polymer brush coating has been proposed. This model is composed of an anti-adhesive molecule that repels bacteria, an antimicrobial peptide that kills bacterial upon contact, and a substance containing arginine-glycine-aspartate that enhances tissue integration [71]. Several other technologies for multifunctional surfaces have been proposed and tested [107,255,256]. Because none of these coating methods can address all criteria defined for anti-infective surface treatment of medical devices intended for long-term usage in orthopaedic surgery (see part 1.4) it has been difficult to implement experimental and preclinical studies.

**Figure 7:**
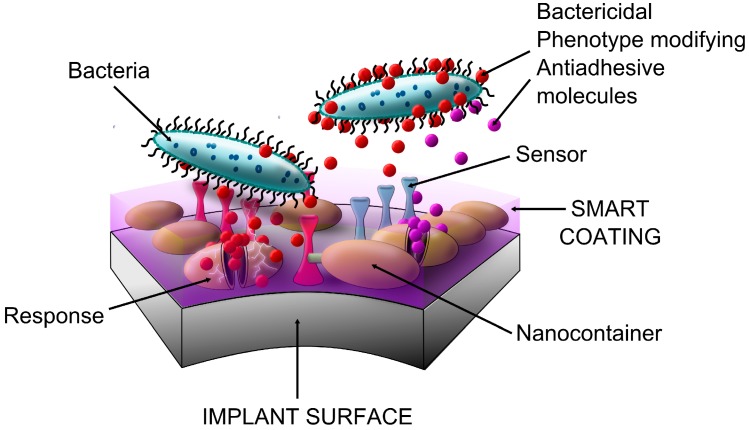
Smart coatings continuously screen the local effective space using molecular receptors specific to a surrounding target’s signals. Stimulation of target-sensitive receptors triggers a cascade of events resulting in outflow of specific substance(s) that affect the target.

Development of multifunctional, self-responsive, and self-repairing biomaterials is becoming one of the most intense areas of anti-infective translational research. “Smart coatings” are designed to be sensitive and responsive to a variety of stimuli such as bacteria [127,129,133]. It is anticipated that these coatings respond intelligently to signaling based on how they are prepared (Figure 7). Accordingly, smart coatings should possess synergistic passive and active functionalities (e.g., anticorrosive, homeostatic, antiosteoclastic, antibacterial, antifungal). Some of the challenges encountered during development of these smart coatings have included: survivorship during the implant-coating manufacturing process, non-adverse reactions to the smart coatings themselves *in vivo*, mechanical resistance, and preservation of intended functionalities throughout the life of the device [128,133,257,258].

A critical component of a smart micro-device is a sensor unit sensitive enough to detect even the most minute microbial-associated signals. Development of nanocontainers with sensitive shells, high loading capacity, and good affinity for the coating matrix has also been challenging [259,260]. Nanocontainer shell entryway signals include pH, temperature, as well as mechanical, chemical, and electrical (electrochemical) changes in the peri-implant effective space [132,133,259]. Stimulation should lead to opening of the nanocontainers and release of specific substance(s).

## 4. Translation of Anti-Infective Coatings into the Clinical Practice

Examination of global grants and published studies on this topic suggests a striking discrepancy between proposed strategies of antibacterial surface treatment and ultimate completion of *in vitro* and *in vivo* experimentation. In fact, we believe that very little progress has actually been made in the translation of the aforementioned modalities into clinically useful technologies. Barriers to translational medicine in this arena are most likely related to economic, medicolegal, and biotechnological issues. Concerns about the long-term durability of such new implants as compared to traditional implants are also realistic. Leaders in this field have recently proposed that in order for some of these obstacles to be overcome we must improve efficiency and effectiveness amongst all partners involved. Only by improving collaborative efforts amongst governments, regulatory agencies, industry leaders and health care payers will patients benefit from these technologies [261]. While pressures exist worldwide to diminish the incidence of PJIs, surprisingly there is not a single large clinical study examining the role of broad-range implementation of implants containing antibacterial surface treatments.

## 5. Conclusions

There is no doubt that prevention is the best response to the growing problem of orthopaedic implant infections. Research in the field of antibacterial surface treatment has demonstrated *in vitro* and *in vivo* effectiveness of several potentially promising technologies. Some interfere with bacterial adhesion and with the initial phases of the biofilm formation. Others exhibit direct antibacterial properties. Strategies incorporating nanopatterning and other nanotechnologies have also shown great promise. In the future, multifunctional smart surfaces could open new avenues for prevention of bacterial attachment while simultaneously enhancing healing and restoration of tissue homeostasis. Issues related to the mechanical properties of these technologies and the potential for detrimental side effects such as toxicity and interference with osseointegration require further investigation. It is of utmost importance to realize, however, that some of the aforementioned technologies have already shown strong enough evidence of antibacterial efficacy, safety, and endurance. The time is here for more efficient development and testing of these technologies in the clinical setting.
